# Is the impact of hospital performance data greater in patients who have compared hospitals?

**DOI:** 10.1186/1472-6963-11-214

**Published:** 2011-09-09

**Authors:** Ingrid B de Groot, Wilma Otten, Harm J Smeets, Perla J Marang-van de Mheen

**Affiliations:** 1 Department of Medical Decision Making, Leiden University Medical Center, The Netherlands; 2 TNO Quality of life, BU Prevention and Care, Section Health Promotion, Leiden, The Netherlands; 3 Department of Surgery, Bronovo Hospital, The Hague, The Netherlands

## Abstract

**Background:**

Public information on average has limited impact on patients' hospital choice. However, the impact may be greater in consumers who have compared hospitals prior to their hospital choice. We therefore assessed whether patients who have compared hospitals based their hospital choice mainly on public information, rather than e.g. advice of their general practitioner and consider other information important than patients who have not compared hospitals.

**Methods:**

337 new surgical patients completed an internet-based questionnaire. They were asked whether they had compared hospitals prior to their hospital choice and which factors influenced their choice. They were also asked to select between four and ten items of hospital information (total: 41 items) relevant for their future hospital choice. These were subsequently used in a hospital choice experiment in which participants were asked to compare hospitals in an Adaptive Choice-Based Conjoint analysis to estimate which of the hospital characteristics had the highest Relative Importance (RI).

**Results:**

Patients who have compared hospitals more often used public information for their hospital choice than patients who have not compared hospitals (12.7% vs. 1.5%, p < 0.001). However, they still mostly relied on their own (47.9%) and other people's experiences (31%) rather than to base their decision on public information. Both groups valued physician's expertise (RI 20.2 [16.6-24.8] in patients comparing hospitals vs. 16.5 [14.2-18.8] in patients not comparing hospitals) and waiting time (RI 15.1 [10.7-19.6] vs. 15.6 [13.2-17.9] respectively) as most important public information. Patients who have compared hospitals assigned greater importance to information on wound infections (p = 0.010) and respect for patients (p = 0.022), but lower importance to hospital distance (p = 0.041).

**Conclusion:**

Public information has limited impact on patient's hospital choice, even in patients who have actually compared hospitals prior to hospital choice.

## Background

Information on performance of hospitals is increasingly available within the public domain in various European countries and worldwide [1], and includes information provided by hospitals and information from previous patients. One of the purposes of this information is to inform consumers about the variation between hospitals so that they can use this information to make informed choices.

The current literature shows that consumers indeed want hospital performance information [2] and that the expected social trend is that use of hospital information will increase over time [3]. However, the current literature also shows that this information only has a limited impact on consumers' choices [4,5]. Part of the limited impact may be that only a small group of patients have looked up and compared hospital information. Various studies showed that only a small group of patients looked up information to compare hospitals, and that the impact on decision making was small for the total group of patients [6,7]. It is likely that the impact on decision making is greater in consumers who have actually compared hospitals since one needs to have looked at information for it to have an effect. It is important to assess whether the impact of public information is greater in consumers who have compared hospitals, since this would suggest it may be possible to enlarge the impact of available information on decision making in some groups. If the impact is similarly small in both groups, then other factors may be more important in decision making and we may have to adjust our expectations about the potential impact of public information as suggested by Marshall et al. [8].

If the impact of public information is greater in consumers who have compared hospitals it is important to explore differences between the two groups. There may be several reasons why people have not compared hospitals. First, consumers may not have a need for comparative information because they always go to the same hospital, so they may only look for information for their preferred hospital [3] or do not trust or understand the provided information [5,9,10]. These reasons may be difficult to influence. Second, consumers may not have compared hospitals because they could not find the information they consider important. In other words, if we would provide all consumers with all the available public information so that they do not have to find the information themselves, to what extent do consumers who have not compared hospitals consider other information important than consumers who have compared hospitals? If they consider other information important, then we may look for reasons why consumers have not found the information, which does not play a role if they consider the same information important.

The present study therefore aims to assess the extent to which patients who have compared hospitals, more often based their hospital choice on public information, and focus on different information than patients who have not compared hospitals. This can be viewed as a first step to assess whether it may be possible to increase the impact of public information in some groups.

## Methods

### Study population

The study population consisted of a group new surgical patients from three hospitals (a university hospital, a general teaching hospital, and a general non-teaching hospital) in the Western region of the Netherlands. They were included only if they visited the surgical outpatient clinic for the first time, as identified by a specific code in the hospital information system. They thus did not have any experience with the surgical department, but may have previous experience with other departments of the hospital.

### Questionnaire

The questionnaire consisted of four parts. The first part included questions about visiting alternative hospitals, the factors influencing their current hospital choice, the awareness of the available hospital information to compare hospitals and the knowledge where to find hospital information. The second and third part of the questionnaire included an Adaptive Choice-Based Conjoint (ACBC) analysis (see also heading methods). Within the ACBC, patients were first asked to select four to ten attributes they would use for future hospital choice from the list of 41 attributes (selection phase in the second part of the questionnaire). Then, in the third part of the questionnaire, the selected hospital attributes were used to present the respondents with different hospitals. For each hospital the respondent had to indicate whether he/she would consider this hospital for treatment or not (screening phase). Only the considered hospitals are taken forward into a final step to identify the overall best hospital (choice phase).

The hospitals compared in the ACBC are not actual hospitals with their data but fictive hospitals designed to assess the importance of hospital information without any distortion being introduced due to naming the hospitals. In the ACBC all participants were presented with all the information available in the public domain, so that they did not have to find this information by themselves. In this way we could assess whether patients who have compared hospitals consider other information important than patients who have not compared hospitals without this being influenced by difficulty finding the information or reputation associated with certain hospital names. In the last part patients were asked for demographic characteristic like age, gender and education.

### Selection of attributes

Hospital attributes and their different levels were selected from available Dutch websites, so that realistic choices between hospitals were presented to patients, closely resembling the actual Dutch situation in which patients may compare hospitals based on public performance data. To identify websites, we searched for hospital information available in the public domain with keywords (e.g. choosing a hospital, performance indicators) and expert advice.

The following websites contained relevant information regarding hospital characteristics and variation between hospitals: http://www.kiesbeter.nl, http://www.independer.nl, http://www.prismant.nl, http://www.nfu.nl, http://www.centrumklantervaringzorg.nl, http://www.rivm.nl, http://www.ziekenhuizentransparant.nlhttp://www.mediquest.nl, http://www.nvz-ziekenhuizen.nl. The information collected by the Netherlands Health Care Inspectorate (NHI) was also presented on these websites, as well as other hospital information. We only included data about hospital attributes if data were available on variation between hospitals. In addition, information had to apply to all surgical patients rather than disease or surgery-specific information, to be relevant for most of the participants.

A part of the information is based on experiences of previous patients, systematically collected using the Consumer Quality-index (CQ-index) hospital admission [11]. In the CQ-index patients are asked to evaluate the quality of care they received during their hospital stay using a scale with grades from zero to ten. This grading system is the same as the Dutch education report card grading system ranging from 0-10, used throughout the school years and in further education in the Netherlands. A grade below 6 is considered insufficient quality of care, consistent with the education system in which a student would fail a test or exam.

### Methods

The study was designed as an experimental choice study, in which we developed an internet-based questionnaire including an Adaptive Choice-Based Conjoint (ACBC) analysis. The questionnaire was developed using SSI Web from Sawtooth Software. Standard CBC is an effective method for understanding how consumers choose between different products or services (in this case hospitals). Simply asking individuals to rate or choose their preferred item from a list may yield no more information than that they want all the benefits and none of the costs. Instead, in a choice experiment respondents are forced to make a trade-off between two or more options. We have previously shown this to be a feasible approach in surgical patients [12]. Recently, Sawtooth Software developed a new approach called Adaptive CBC (ACBC) [13].

The main advantage of ACBC is that each respondent first selects hospital characteristics (attributes) from a list that are relevant for him/her personally when choosing a hospital, so that attribute sets may differ between individuals. In a CBC on the other hand, the investigator chooses a fixed number of attributes and presents these to respondents with the possibility that some of the attributes are not relevant for the respondent. Consequently, the results of a CBC may not accurately reflect which information is important when consumers choose a hospital if they are not able to select the attributes relevant for their situation.

The hospital attributes selected by the respondent in the ACBC were then used to present the respondent with different hospitals. For each hospital the respondent had to indicate whether he/she would consider this hospital for treatment or not. Forty-one hospital attributes were selected for the questionnaire. The order in which attributes were presented to patients was randomized to prevent that first presented attributes received more attention and thereby a higher importance. Based on empirical data, three levels were constructed for each attribute given the minimum, median and maximum estimate. The only exception was the attribute 'type of hospital', for which two levels was constructed (academic hospital and general hospital).

During the screening phase the Sawtooth software notices when a respondent systematically avoids an attribute level or if the respondent expresses interest in only one attribute level. The respondent is asked whether that level is completely unacceptable or an absolute requirement ('must have'). In case the respondent identifies unacceptable levels or 'must have' levels, then all further 'hospitals' that are shown from that point onwards will satisfy those requirements. In this screening phase respondents were presented with a maximum of eight screening tasks.

Considered hospitals are taken forward into a final step (choice phase) to identify the overall best hospital. Respondents had to choose one out of three hospitals, with a maximum of eight choice tasks (the exact number depending on the number of attributes selected and the use of 'unacceptables' and 'must haves').

In the ACBC the respondent is presented with relevant comparisons only through the preselection of attributes and the use of 'unacceptables' and 'must haves' in the screening phase. At the same time fewer comparisons can be presented. The ACBC questionnaire therefore is experienced as a realistic and personalized questionnaire, which is more engaging. Moreover, ACBC requires smaller sample sizes than standard CBC, because more information is captured from each individual [13,14]. Therefore, this method is even more effective than the previously used CBC [15] to assess which hospital information determines consumers' hospital choice, particularly when there is conflicting information regarding which hospital is best, since it forces consumers to choose which attribute takes precedence over others.

From the choices made by the respondent, the individual's utility for each attribute level can be inferred. Based on the ranges of these utilities the relative importance (RI) of the attributes can be determined (see heading analysis).

### Procedure

The patients received written information about this study and a response form before visiting the outpatient clinic. On this form patients could indicate if they wanted to participate or not. If not, they were asked for their reason of non-participation. At their first visit to the outpatient clinic they were asked to hand in the response form, to ensure that we received all response forms.

All patients received the link to the website and a Response ID number by email. If the questionnaire was not filled in after ten days, a reminder was sent. The study was approved by the Medical Ethical Committee of the Leiden University Medical Center.

### Analyses

Hierarchical Bayes (HB) estimation was used to estimate the individual utilities for each attribute level using the ACBC Sawtooth software [12]. This method uses an iterative process, along with information from the patients, to estimate the utilities for each subject. Using Gibbs sampling, attribute level utilities are estimated that best fit each patient, borrowing information from other patients to stabilize the model [15].

The RI of each attribute was used as a measure of the influence of each attribute on decision making. The RI of each attribute (A) for each individual is calculated according to:

$$\text{RI}=\frac{\text{Range }{\text{A}}_{i}}{\underset{i=1\;\text{to}\;n}{\sum }\text{Range }{\text{A}}_{i}}\times 100$$

Where Range A_i _is the difference between the highest and lowest utility for the i^th ^attribute, n is the number of attributes and A_i _is the i^th ^attribute.

Based on the choice tasks in the choice phase, after preselection of must haves and unacceptables, the utilities of the attribute levels and the RIs of the attributes are calculated for each individual respondent. The sum of all RIs is thus 100 for each individual. Must haves result in more extreme utilities indicating a stronger preference, and thereby in a higher RI, consistent with the notion that the respondent is saying that this attribute is very important. So a high RI indicates a stronger preference of one attribute over another level, relative to other attributes and thus indicates a high impact on decision making, whereas a low RI indicates a low impact on decision making. When an attribute is not selected by a respondent the RI of this attribute is set to zero for this individual.

To assess which attribute on average is most and least influential in this population, as well as the order in between, we calculated the average RI of an attribute over all respondents with its 95% confidence interval.

The Goodness-of-fit was determined by calculating the Root Likelihood (RLH) [16]. This can be compared to the null RLH i.e. the RLH expected by chance, defined as one divided by the number of alternatives, which is 0.33 in this study given that three hospitals are presented to patients.

Respondents and non-respondents were compared on possible differences in demographic variables. Differences between groups were tested using chi-square tests or using Fisher's exact test in case cells had an expected count less than five.

The study population was divided into patients who compared hospitals (regardless whether patients have compared two or more options) and patients who did not compare hospitals. This was based on a single question by which patients could indicate whether they had compared hospitals for their current hospital choice, or not. We assessed whether patients who compared hospitals differed in age, gender and educational level from patients who did not compare hospitals. We distinguished two age groups (< 65 years and 65+ years) and three educational level groups: basic education (no or only primary education), intermediate education (pre-vocational secondary education, senior secondary vocational training, senior secondary general education or university, pre-university education) or high education (higher professional education or university (Master, Bachelor or PhD)).

Patients not comparing hospitals still might have looked up public information (e.g., only for their preferred hospital) or may have deliberately chosen to go to the same hospital they always go to. With additional questions patients were therefore asked which factors influenced their choice and whether they looked up public information. Based on this information we assessed whether patients who compared hospitals more often used public information for their hospital choice than patients who did not compare hospitals, and differed in other factors influencing their current hospital choice.

Furthermore, we assessed whether the two groups considered other hospital information as most important, by investigating whether the RI of attributes differed between the two groups of patients. We first compared the average RI between the two groups using univariate linear regression analysis. Second, we assessed whether differences between the two groups remained after adjusting for age, gender, level of education using multivariate linear regression analyses. In all statistical analyses a p-value less than 0.05 was considered statistically significant.

## Results

643 patients were included of which 461 (71.7%) patients consented to participate and 337 (52.4%) patients had complete data. The most important reason given for non-participation was not having a computer with internet connection (43.2%). The participating patients did not differ in gender (X^2 ^= 1.36, p = 0.24), but were younger (X^2 ^= 38.93, p < 0.01) than non-participating patients. Level of education was not available for non-participating patients, so that a comparison was not possible. Compared to the general Dutch population [17], our study population included relatively higher educated individuals, but was similar with respect to age and gender (Table 1).

**Table 1:** Demographic characteristics of the study population and the general Dutch population

	Study population			Dutch population
	**Patients who have compared hospitals** **(n = 71)**	**Patients who have not compared hospitals** **(n = 266)**	**Test of difference**	

Gender				
Male %	49.3%	50.4%	X^2 ^= 0.03, p = 0.87	49.5%

Age				
< 65%	83.1%	80.5%	X^2 ^= 0.26, p = 0.61	84.7%

Educational level				
Basic %	2.9%	2.7%	X^2 ^= 0.09, p = 0.99	19.0%
Intermediate %	56.5%	56.4%		54.2%
High %	40.6%	40.9%		26.3%

General Dutch population: www.CBS.nl (Statistics Netherlands)

From the participating patients, only 71 (21.1%) patients had compared hospitals. No differences were found between patients who did and did not compare hospitals regarding gender, age and level of education (Table 1). At least half of the patients indicated that they were aware of information on hospitals to compare hospitals and most of them knew were to find this information. However few patients actually looked up this information to compare hospitals (Table 2).

**Table 2:** Differences between patients who have and have not compared hospitals regarding factors influencing hospital choice

		Patients who have compared hospitals (n = 71)	Patients who have not compared hospitals (n = 266)	Test of difference	
				
				X^2^	p
Did you deliberately choose for this hospital?	Yes	67 (94.4)	198 (74.4)	13.25	**< 0.001**

What issues have played a role in your choice?	Own previous experience	34 (47.9)	115 (43.2)	0.49	0.48
	Experience of people from my environment	22 (31.0)	35 (13.2)	12.68	**< 0.001**
	Advice from the general practitioner	21 (29.6)	94 (35.3)	0.83	0.36
	Public information in the media	9 (12.7)	4 (1.5)	18.86	**< 0.001**
	None	0 (0.0)	3 (1.1)	0.81	1.00

Patients have the right to choose their own hospital. Did you know that you can choose in which hospital you want to be treated?	Yes	70 (98.6%)	250 (94.0%)	2.48	0.14

Are you aware of information on hospitals, which you can use to compare hospitals?	Yes	36 (50.7%)	162 (60.9%)	0.41	0.12
If Yes: Do you know were you can find this information?	Yes	28 (77.8%)	110 (67.9%)	1.36	0.24

Did you look up information on more than one hospital?	No, I did not look up information	32 (45.1)	195 (73.3)		
	No, I only looked up for information regarding the hospital I visited	12 (16.9)	36 (13.5)	28.58	**< 0.001**
	Yes, I looked up information regarding other hospitals, I compared the hospitals and then made a choice	13 (18.3)	11 (4.1)		
	Other	14 (19.7)	24 (9.0)		

Significant differences are presented in bold. Multiple answers were possible in some questions.

Patients who compared hospitals more often chose deliberately for the current hospital (p < 0.05) and more often relied on experiences from other people and on public information in the media compared to patients who did not compare hospitals (p < 0.05) (Table 2). However, their choice still was mainly based on their own previous experience with other departments of the hospital (47.9%) or the advice of their general practitioner (29.6%), as it did in patients who did not compare hospitals (43.2% and 35.3% respectively). These results are not explained by e.g. more patients with higher education in the group comparing hospitals, given that the groups did not differ in age, gender or educational level.

### Relative importance of the attributes in the total population

'Report card grade regarding physician's expertise' had the highest relative importance (RI) followed by 'Waiting time for appointment at the outpatient clinic' (Table 3). This information clearly had a higher RI than the next important characteristics 'Waiting time for surgery' and 'Positive judgment about physician communication', given the non-overlapping confidence intervals. These four attributes were most important in both men and women, in patients aged below 65 years and the two highest educational groups (data not shown).

**Table 3:** Selected attributes, levels and relative importance (RI) between patients who have and have not compared hospitals

Information on hospital	Levels	All patients (n = 337) RI [95%CI]	Patients who have compared hospitals (n = 71) RI [95%CI]	Patients who have not compared hospitals (n = 266) RI [95%CI]	Univariate test of difference β [95% CI]
Report card grade regarding physician's expertise	6.9 vs. 7.7 vs.8.6	17.29 [15.22-19.35]	20.17 [15.57-24.77]	16.52 [14.20-18.83]	3.65 [-1.40-8.71]
Waiting time for outpatient clinic appointment	1 week vs. 4 weeks vs. 7 weeks	15.46 [13.40-17.53]	15.12 [10.66-19.59]	15.55 [13.21-17.90]	-0.43 [-5.51-4.65]
Waiting time for surgery	1 week vs. 3 weeks vs. 5 weeks	7.83 [6.63-9.04]	6.97 [4.44-9.49]	8.06 [6.69-9.44]	1.10 [-4.05-1.86]
Positive judgment regarding physicians communication	49% vs. 58% vs. 68%	7.70 [6.67-8.73]	7.40 [5.31-9.49]	7.79 [6.60-8.97]	-0.38 [-2.91-2.14]
Surgery with textbook outcome	72% vs. 81% vs. 90%	4.23 [3.32-5.15]	5.73 [3.64-7.82]	3.83 [2.81-4.85]	1.90 [-0.35-4.14]
Attention to pain management	10% vs. 55% vs.100%	3.66 [2.74-4.58]	2.87 [1.03-4.72]	3.87 [2.81-4.93]	-1.00 [-3.25-1.26]
Positive judgment regarding treatment explanation	52% vs. 63% vs. 71%	3.63 [2.95-4.31]	3.51 [2.32-4.70]	3.66 [2.86-4.47]	-0.15 [-1.82-1.53]
Distance to hospital	5 km vs. 10 km vs. 20 km	3.59 [2.76-4.43]	1.91[0.73-3.09]	4.04 [3.03-5.05]	**-2.13 [-4.17;-0.09]**
Type of hospital	academic or non-academic hospital	3.30 [2.53-4.07]	3.30 [1.96-4.64]	3.30 [2.39-4.21]	0.00 [-0.189-1.89]
Positive judgment regarding nurses' communication	43% vs. 54% vs. 66%	2.66 [2.13-3.18]	2.18 [1.12-3.24]	2.78 [2.18-3.39]	-0.60 [-1.89-0.69]
Sufficient privacy and ability to participate in decisions	32% vs. 39% vs. 46%	2.56 [2.05-3.07]	1.76 [0.94-2.58]	2.78 [2.17-3.38]	-1.02 [-2.26-0.23]
Complications	6% vs. 14% vs. 23%	2.56 [1.76-3.36]	2.38 [1.03-3.72]	2.61 [1.66-3.56]	-0.23 [-2.19-1.73]
Travel time to hospital	10 min vs. 20 min vs.30 min	2.40 [1.86-2.95]	1.89 [0.43-3.34]	2.54 [1.97-3.12]	-0.66 [-1.99-0.68]
Report card grade regarding the care provided by physicians	7.7 vs. 8.1 vs.8.6	2.38 [1.92-2.85]	2.39 [1.53-3.24]	2.38 [1.84-2.93]	0.00 [-1.14-1.15]
Report card grade regarding the quality of the care	6.6 vs. 7.5 vs. 8.4	2.07 [1.62-2.52]	2.65 [1.64-3.65]	1.91 [1.41-2.42]	0.74 [-0.38-1.84]
Overall report card grade hospital	7.3 vs. 7.9 vs. 8.5	1.74 [1.32 -2.16]	1.34 [0.68-2.00]	1.85 [1.35-2.35]	-0.51 [-1.53-0.51]
Percentage of patients who would recommend the hospital	29% vs. 51% vs.74%	1.49 [1.02 -1.95]	1.71 [0.52-2.89]	1.43 [0.92-1.93]	0.28 [-0.86-1.43]
Report card grade regarding the care provided by nurses	7.6 vs. 8.0 vs. 8.5	1.44 [1.10-1.78]	1.22 [0.56-1.88]	1.50 [1.11-1.90]	-0.28 [-1.12-0.55]
Positive judgment regarding pain management	49% vs. 61% vs. 74%	1.25 [0.82-1.68]	1.59 [0.40-2.79]	1.16 [0.71-1.60]	0.44 [-0.61-1.49]
No problems with coordination between health care providers	75% vs. 84% vs. 94%	1.17 [0.81-1.52]	0.84 [0.26-1.43]	1.26 [0.83-1.68]	-0.41 [-1.28-0.46]
Positive judgment regarding medication explanation	31% vs. 48% vs. 66%	1.15 [0.76-1.54]	0.71 [0.14-1.28]	1.27 [0.80-1.74]	-0.56 [-1.52-0.39]
Report card grade regarding information provision to the patient	6.4 vs. 7.0 vs.7.7	1.00 [0.71-1.29]	1.53 [0.79-2.28]	0.86 [0.55-1.17]	0.67 [-0.04-1.38]
Positive judgment regarding patient safety	48% vs. 57% vs. 66%	0.99 [0.63-1.36]	0.91 [0.25-1.56]	1.02 [0.59-1.45]	-0.11 [-1.01-0.79]
Positive judgment regarding personal treatment and reception by professionals	92% vs. 95% vs.99%	0.82 [0.57-1.08]	1.09 [0.45-1.73]	0.75 [0.48-1.03]	0.33 [-0.29-0.96]
Report card grade regarding reception when admitted	7.3 vs.7.8 vs. 8.3	0.82 [0.57-1.08]	0.36 [0.03-0.68]	0.31 [0.14-0.48]	0.05 [-0.32-0.42]
Positive judgment regarding the hospital room and food	37% vs. 49% vs.61%	0.74 [0.43-1.04]	0.52 [0.06-0.99]	0.79 [0.42-1.17]	-0.27 [-1.03-0.49]
Positive judgment regarding the content of the interview at admission	57% vs.64% vs.71%	0.69 [0.45-0.94]	0.69 [0.21-1.16]	0.69 [0.41-0.98]	-0.01 [-0.61-0.60]
No problems with hospital accessibility	48% vs. 71% vs. 94%	0.66 [0.38-0.94]	0.41 [0.02-0.79]	0.72 [0.38-1.07]	-0.32 [-1.01-0.37]
Mean duration of hospital admission	5 days vs. 7 days vs. 9 days	0.66 [0.39-0.93]	0.37 [0.03-0.71]	0.73 [0.40-1.06]	-0.36 [-1.03-0.30]
Admission in daycare	15% vs. 29% vs. 43%	0.64 [0.40-0.89]	0.61 [-0.02-1.22]	0.65 [0.39-0.92]	-0.04 [-0.64-0.55]
Report card grade regarding respect for patients	6.8 vs. 7.5 vs. .8.2	0.58 [0.31-0.85]	1.19 [0.11-2.27]	0.42 [0.24-0.61]	**0.77 [0.11-1.42]**
Availability of electronic patient data	Score 6 vs. Score 8 vs. Score 10	0.55 [0.27-0.83]	0.45 [-0.22-1.13]	0.57 [0.26-0.88]	-0.12 [-0.80-0.57]
Report card grade regarding the quality of discharge and after care	6.1 vs. 6.9 vs. 7.8	0.50 [0.26-0.74]	0.85 [0.12-1.58]	0.41 [0.17-0.65]	0.44 [-0.15-1.03]
Wound infections	1% vs. 2% vs. 4%	0.42 [0.23-0.61]	0.90 [0.28-1.52]	0.30 [0.12-0.47]	**0.61 [0.14-1.07]**
Re-operation or readmission	6% vs. 9% vs.12%	0.39 [0.15-0.63]	0.85 [0.15-1.54]	0.27 [0.02-0.52]	0.58 [-0.20-1.17]
Size of hospital (number of beds)	150 vs. 500 vs. 1000 beds	0.31 [0.14-0.49]	0.37 [-0.03-0.76]	-0.30 [0.10-0.50]	0.07 [-0.37-0.50]
Death during admission	0.5% vs. 1% vs. 2%	0.31 [0.12-0.50]	0.67 [0.11-1.22]	0.22 [0.03-0.41]	0.45 [-0.20-0.91]
Cancelled surgeries within 24 hours before the scheduled date	0.5% vs. 3% vs. 6%	0.28 [0.11-0.44]	0.00 [0.00-0.00]	0.35 [0.14-0.56]	-0.35 [-0.76-0.06]
Positive judgment regarding information during hospital discharge	67% vs. 76% vs. 85%	0.27 [0.15-0.39]	0.13 [-0.08-0.29]	0.31 [0.16-0.46]	-0.18 [-0.48-0.12]
Access to information on medication use at the outpatient clinic	0% vs. 50% vs. 100%	0.18 [0.04-0.31]	0.41 [-0.08-0.91]	0.12 [0.01-0.22]	0.30 [-0.03-0.63]
Pressure sores	1% vs. 6% vs. 11%	0.10 [-0.01-0.21]	0.07 [-0.07-0.20]	0.11 [-0.02-0.24]	-0.04 [-0.30-0.22]
		**Model fit: Root Likely Hood: 0.617**			

Significant differences are presented in bold

In the lowest educational level group 'Waiting time for outpatient clinic appointment' had the highest RI (20.83 [5.66-35.99]), followed by 'Waiting time for surgery' (15.12 [0.68-29.55]), 'Physicians communication' (7.70, [2.07-13.33]) and 'Operation with textbook outcome' (5.79 [-1.85-13.44]). In patients aged above 65 years 'Report card grade regarding physician's expertise' (12.84 [8.12-17.55]) had the highest RI, followed by 'Positive judgment about physician communication' (9.75 [7.24-12.27]), 'Attention to pain management' (5.78 [2.86-8.30]) and 'Waiting time for appointment at the outpatient clinic' (5.38 [3.02-7.75]).

In the total population, the attributes 'Access to information about medication use at the outpatient clinic' (0.18 [0.04-0.31]) and 'Pressure sores' (0.10 [-0.01-0.21]) had the lowest RI (Table 3).

### Differences between patients who did and did not compare hospitals

Both patients who did and did not compare hospitals assigned the greatest importance to the same four attributes (Table 3). However, patients comparing hospitals assigned greater importance to 'Wound infections' and 'Report card grade regarding respect for patients' than patients who did not compare hospitals. At the same time, they assigned lower importance to 'Hospital distance' (Table 3).

After adjustment for age, gender and educational level, only differences in 'Wound infections' (β = 0.06 [0.11-1.06]) and 'Report card grade regarding respect for patients' (β = 0.75 [0.07-1.42]) remained.

## Discussion

This study has shown that patients who have compared hospitals more often based their hospital choice on public information than patients who have not compared hospitals, but still their decision was mostly based on their own and other people's experiences. Both patients who have compared hospitals and patients who have not compared hospitals value the same public hospital information as most important, that is information regarding physician's expertise, waiting time and communication. However, patients who have compared hospitals more often focus on information on wound infections and report card grade regarding respect for patients and they seem to be prepared to travel further to visit their hospital of choice, given the lower importance assigned to hospital distance.

Our results may have been biased due to the selection of participating patients. This affects our results when these patients choose differently than the non-participants. The participating patients were younger than the non-participating patients and our study population was higher educated than the Dutch population, most likely explained by the use of an internet-based questionnaire. The availability of a computer and internet tends to be higher in individuals from higher socioeconomic groups and in younger individuals [17]. In the group of less educated individuals, waiting time was considered more important than in the group of individuals with higher educational levels. However, this concerned a small number of less educated individuals so that we have to be careful with our conclusions regarding the effect this may have on the outcomes of our study. Assuming that the higher importance of waiting time is true in a larger group of less educated individuals, then the importance of waiting time may have been underestimated in our study due to selective participation. Similarly, in patients aged above 65, physicians' communication and attention to pain management were considered more important than in patients aged younger than age 65. The importance of physicians' communication and attention to pain management may have been underestimated in this study, due to selective participation of younger patients.

Regarding the type of public information that is considered important, both patient groups focus on information regarding physician's expertise, waiting time and physician's communication when choosing a hospital. Other studies also showed that doctor communication was the most important item that influenced the choice of hospital besides friendly staff [2,18,19]. Waiting time was also considered most important in a previous study among surgical patients [3]. These results regarding the type of information are thus consistent with other studies. Previous research also showed that information on outcomes of care hardly influenced patients' choice of health care provider [6,7], but no comparison was made between patients who have and have not compared hospitals and whether they choose differently. The present study thus adds that patients who have compared hospitals consider the same hospital information important as patients who have not compared hospitals, so that we can assume that even in the group actually comparing hospitals, public information only has a minor impact on their hospital choice.

A minority of our study population used public information to compare hospitals. This is consistent with the study of Schwartz et al. who found that few respondents (11%) looked up information to compare hospitals [7]. In the current study patients mostly relied on their own experiences or experiences of other people with the hospital and on the advice of the general practitioner when choosing a hospital. These results seem to be similar to that of other studies showing that comparative consumer information is not the primary information consumers base their choice on, but that they rely mostly on advice of their physician, family or friends [9,20,21].

There may be several explanations for our findings that public information is hardly used to compare hospitals and therefore has little influence on decision-making. These can be found in either not searching for information and/or not using this information to compare hospitals. First, many patients rely on the advice of their general practitioner, possibly assuming that their physician uses hospital information to recommend a hospital as suggested by Schwartz et al. [7], so that they are less likely to search for information to compare hospitals by themselves. Second, as suggested in the literature, patients are often unaware that information is available [4]. Only half of our study population indicated that they were aware that public hospital information is available and even a smaller proportion of the patients knew were to find this information. This situation may change in the future, as consumers become more familiar with the internet in general and with websites providing comparative consumer information. Third, patients may be aware of hospital information, but do not use this information to compare hospitals since they always go to the same hospital. It is possible that they only search for information for their preferred hospital. In our study population only a small part (14-15%) checked information regarding the hospital they visited, so that this does not seem to be the entire explanation. Another possibility is that patients do know where to find information, but do not understand, trust or believe this information. If consumers do not understand certain information they are more likely to ignore it or to consider it as unimportant and consequently do not use it to compare hospitals [10]. This would explain why medically oriented quality of care information, like percentage of complications or percentage of wound infections are valued lower than measures on report card grade, which is familiar to everybody since these are used throughout the educational system. It is also possible that the information is not easily available or that patients do not like the way the information is presented on the websites. Studies indicate that the way information is presented affects whether patients understand and use this information [10]. Currently, consumers have to visit different websites for relevant information and have to group the available information on their own. A more structured presentation of the information (e.g. in groups) may facilitate finding the information and thereby the chances that it is used in patients decision making. Further research should explore how patients' decision making is supported in the best possible way.

## Conclusions

Even in patients who used public information to compare hospitals, hospital choice was mostly based on their own and other people's experiences. Patients who have compared hospitals prior to their visit focus on the same public hospital information as patients who have not compared hospitals. Therefore, it seems likely that choosing a hospital is not a rational process based on the cognitive assessment of information. In line with a recent analysis by Marshall & McLoughin [8], we believe that hospital choice may be a more social process in which experiences are important. They often have a strong sense of loyalty and indebtedness, which can transcend hard evidence about performance. For example, a person might decide not to choose highly rated hospital only because their grandmother died there even though CQ-data suggest otherwise. It may still be possible to further increase the impact of public information on decision making, but this probably requires a different type of hospital information. For instance, presenting reviews of other people in combination with a score may better enable consumers to interpret the score themselves, because they get more detailed information on the reasons behind a low or high score, similar to current practice regarding e.g. hotel reviews. People may be more likely to trust and thus use this information in decision making.

## Declaration of competing interests

The authors declare that they have no competing interests.

## Authors' contributions

IBG carried out the study, performed the statistical analyses and interpreted the data, and wrote the first draft of the manuscript. WO and HJS participated in study design, interpretation of the data and writing the manuscript. PJM conceived the study and its design, participated in the statistical analyses and interpretation of the data, and writing the manuscript. All authors have read and approved the final manuscript.

## Pre-publication history

The pre-publication history for this paper can be accessed here:

http://www.biomedcentral.com/1472-6963/11/214/prepub
